# THE STRENGTH OF PARTNERSHIPS IN EMERGENCY RESPONSE AND RESILIENCE: FINDINGS FROM THE VOICES OF OLDER NEW YORKERS

**DOI:** 10.1093/geroni/igae098.1610

**Published:** 2024-12-31

**Authors:** Elana Kieffer

**Affiliations:** The New York Academy of Medicine, New York, New York, United States

## Abstract

New York City is home to over 1.2 million adults age 65+ in approximately 300 square miles. The COVID-19 pandemic and the ongoing climate crisis have presented older New Yorkers with new public health challenges and exacerbated existing ones. The time is now for aging service providers, researchers, and older adults to develop and coordinate efforts to build, maintain, and increase community resilience in the wake of emergencies and disasters. In this session, staff members of the Center for Healthy Aging at The New York Academy of Medicine (NYAM) will describe how older New Yorkers and the professionals who work with them have demonstrated resilience since the pandemic, and provide recommendations on how they can continue to do so, as outlined in “Strengthening Community Resilience: Supporting Older Adults Through Emergency Preparedness And Response In A Post-COVID Era,” published by NYAM in 2023. Guided by the RAND Corporation’s Framework for Community Resilience and NYAM’s previous research on resilience in the wake of Superstorm Sandy, we will discuss the results of this report, including the importance of partnerships, education, program efficiency, and access to resources, all through a strengths-based lens. This presentation will describe these key findings and their potential implications in the context of advocacy and policy change.

